# Serum and Tissue Zinc in Epithelial Malignancies: A Meta-Analysis

**DOI:** 10.1371/journal.pone.0099790

**Published:** 2014-06-18

**Authors:** Jaromir Gumulec, Michal Masarik, Vojtech Adam, Tomas Eckschlager, Ivo Provaznik, Rene Kizek

**Affiliations:** 1 Department of Pathological Physiology, Faculty of Medicine, Masaryk University, Brno, Czech Republic; 2 Central European Institute of Technology, Brno University of Technology, Brno, Czech Republic; 3 Department of Chemistry and Biochemistry, Mendel University in Brno, Brno, Czech Republic; 4 Department of Paediatric Haematology and Oncology, 2nd Faculty of Medicine and University Hospital Motol, Charles University, Prague, Czech Republic; 5 Department of Biomedical Engineering, Faculty of Electrical Engineering and Communication, Brno University of Technology, Brno, Czech Republic; State University of Maringá/Universidade Estadual de Maringá, Brazil

## Abstract

**Background and Objectives:**

Current studies give us inconsistent results regarding the association of neoplasms and zinc(II) serum and tissues concentrations. The results of to-date studies using meta-analysis are summarized in this paper.

**Methods:**

Web of Science (Science citation index expanded), PubMed (Medline), Embase and CENTRAL were searched. Articles were reviewed by two evaluators; quality was assessed by Newcastle-Ottawa scale; meta-analysis was performed including meta-regression and publication bias analysis.

**Results:**

Analysis was performed on 114 case control, cohort and cross-sectional studies of 22737 participants. Decreased serum zinc level was found in patients with lung (effect size = −1.04), head and neck (effect size = −1.43), breast (effect size = −0.93), liver (effect size = −2.29), stomach (effect size = −1.59), and prostate (effect size = −1.36) cancers; elevation was not proven in any tumor. More specific zinc patterns are evident at tissue level, showing increase in breast cancer tissue (effect size = 1.80) and decrease in prostatic (effect size = −3.90), liver (effect size = −8.26), lung (effect size = −3.12), and thyroid cancer (effect size = −2.84). The rest of the included tumors brought ambiguous results, both in serum and tissue zinc levels across the studies. The association between zinc level and stage or grade of tumor has not been revealed by meta-regression.

**Conclusion:**

This study provides evidence on cancer-specific tissue zinc level alteration. Although serum zinc decrease was associated with most tumors mentioned herein, further – prospective - studies are needed.

## Introduction

Zinc(II) plays a role in several intracellular signalling pathways. It is also a cofactor of numerous enzymes [1]. Its dysregulation is present in various cancers. Imbalance of zinc transporters causing intracellular and serum zinc(II) levels alteration was described in prostate and breast cancers. [2]–[5]. Questions were raised whether these associations have clinical applications. Studies focusing on zinc content in biological materials in cancer patients provide inconsistent results. Zinc levels in tumor tissues of prostate [6], liver [7], and lung [8] and its serum levels in breast, lung, stomach, and prostate cancer patients were reviewed previously [9].

We investigated the associations of serum and cellular zinc(II) levels with carcinomas via meta-analysis.

## Methods

### Literature Search

**Figure 1:**
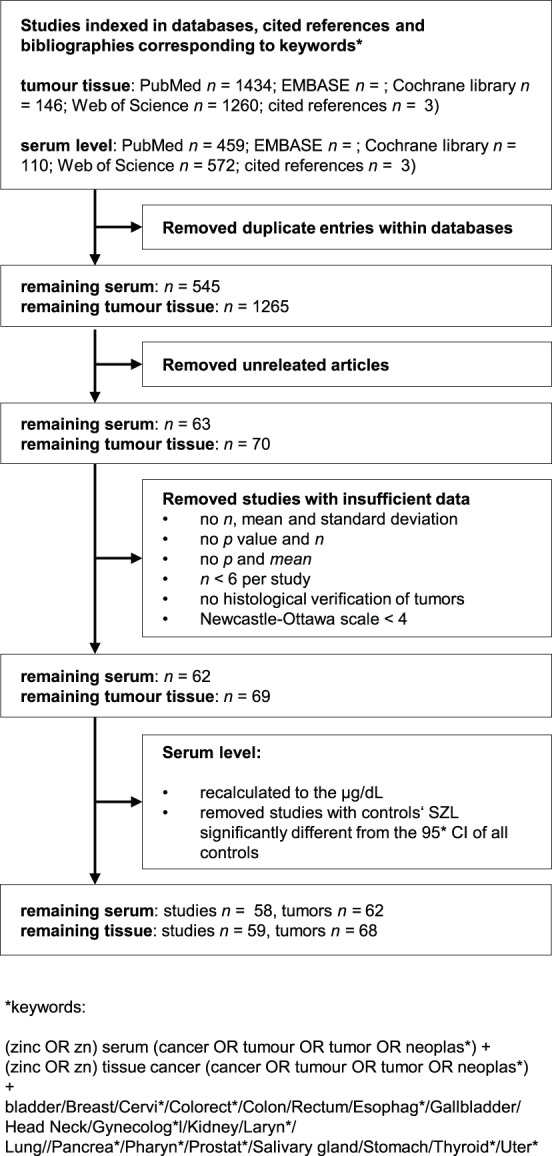
Flow diagram for identification of relevant studies.

Search was performed in Web of science (Science citation index expanded 1945 to April 2013), PubMed (Medline 1968 to April 2013), Embase (1977 to April 2013), and Cochrane Library (CENTRAL 1953 to April 2013); keywords are shown in Figure 1. Moreover, cited references of found articles were analyzed.

### Selection Criteria

Diagram (Fig. 1) shows acquisition process. Among published articles, the search was done for clinical trials, case-control and cohort studies investigating the associations between carcinoma and tissues and serum zinc levels. Because no difference in zinc(II) level between serum and plasma was found [10], both materials were referred as “serum”. Studies with full texts available were included only. Only the studies where the data were displayed in the following ways were accepted: (1) sample size, means and standard deviations, or (2) sample size, means, P values and statistical test type (one- or two-tailed). If similar data were found in more studies by the same group, study with most data was included.

The eligibility of the studies for meta-analysis was evaluated by J.G. and V.A., discrepancies were discussed with R.K and M.M.

### Assessment of Methodological Quality

The quality of studies was assessed by the Newcastle-Ottawa Scale (NOS) [11]. NOS ranges from 0 to 9 stars. The studies with <4 stars were excluded, with >6 stars were considered as high quality; the mean was 5.6 stars.

### Main and Subgroup Analyses

First, differences of the serum and tissue zinc levels between overall tumors and controls were analysed (Table 1). Then, analyses by tumor type, histology, and methodological quality were performed (Table 2). To meet conditions of between-study independence, zinc level was averaged in studies with multiple tumors, forming age groups or detecting gender separately in case of the summary. For comparison of individual tumors, the tumor was taken as a unit of analysis unless violated between-study independence.

**Table 1:** Overall results of meta-analysis by tumor type and statistical model used.

Tumor	No. of studies	Point estimate	95% CI of point estimate	Heterogeneity, I^2^	Model used
Bladder - serum	2	−1.24	−1.77 to −0.71	61.74	random
Breast serum	12	−0.93	−1.68 to −0.17	96.16	random
Breast tissue	15	1.80	1.17 to 2.42	94.89	random
Colorectal serum	5	0.04	−2.57 to 2.64	98.98	random
Colorectal tissue	7	0.37	−0.97 to 1.72	96.23	random
Esophageal serum	4	−2.17	−3.23 to −1.11	86.46	random
Esophageal tissue	3	−1.57	−3.17 to 0.03	94.72	random
Gallbladder - serum	1	−2.31	−2.96 to −1.65		-
Gallbladder - tissue	2	−1.25	−1.73 to −0.77	0.00	fixed
Gynecological serum	3	−0.39	−0.6 to −0.17	49.48	fixed
Gynecological tissue	4	−0.70	−1.85 to 0.45	91.40	random
Head and neck tissue	2	3.11	−5.96 to 12.17	98.75	random
Head Neck serum	5	−1.43	−2.17 to −0.68	77.46	random
Kidney - tissue	4	−2.23	−3.89 to −0.57	89.51	random
Liver - serum	3	−2.29	−5.21 to 0.63	97.64	random
Liver - tissue	7	−8.26	−11.02 to −5.49	98.49	random
Lung - serum	13	−1.04	−1.53 to −0.56	92.94	random
Lung - tissue	6	−3.12	−4.57 to −1.67	96.76	random
Prostate serum	7	−1.36	−1.97 to −0.75	97.93	random
Prostate tissue	12	−3.90	−5.26 to −2.54	94.67	random
Stomach serum	4	−1.59	−3.14 to −0.03	98.24	random
Stomach tissue	3	−0.79	−1.44 to −0.14	60.85	random
Thyroid serum	3	−0.62	−3.04 to 1.79	97.69	random
Thyroid tissue	3	−2.84	−5.39 to −0.29	97.56	random
Overall - serum	58	−1.08	−1.33 to −0.82	96.71	random
Overall - tissue	59	−1.44	−1.93 to −0.95	97.08	random

CI, confidence interval.

**Table 2:** Subgroup analysis by study quality and histological type.

Subgroup	Tumor	Factor	No. of studies	Point estimate (95% CI)	Heterogeneity, I^2^	Model used
**Study quality**						
	Breast serum	high	3	0.22 (−0.08 to 0.51)	0.00	fixed
	Breast serum	low	9	−1.32 (−2.23 to −0.42)	97.06	random
	Breast tissue	high	9	2.33 (1.46 to 3.21)	95.88	random
	Breast tissue	low	6	1.11 (0.08 to 2.14)	91.22	random
	Colorectal tissue	high	2	−0.50 (−2.84 to 1.85)	81.73	random
	Colorectal tissue	low	5	0.80 (−0.84 to 2.43)	96.55	random
	Esophageal serum	high	2	−1.56 (−3.23 to 0.10)	72.04	random
	Esophageal serum	low	2	−2.99 (−4.79 to −1.19)	93.50	random
	Esophageal tissue	high	2	−1.60 (−3.86 to 0.65)	97.29	random
	Esophageal tissue	low	1	−1.52 (−2.56 to −0.47)		-
	Gynecological tissue	high	3	−0.14 (−1.02 to 0.75)	82.33	random
	Gynecological tissue	low	1	−2.49 (−3.31 to −1.66)		-
	Head Neck serum	high	2	−0.88 (−1.26 to −0.49)	3.16	fixed
	Head Neck serum	low	3	−1.59 (−2.08 to −1.10)	86.60	random
	Kidney - tissue	high	2	−1.03 (−1.80 to −0.26)	22.30	fixed
	Kidney - tissue	low	2	−3.12 (−6.49 to 0.25)	96.07	random
	Liver - tissue	high	2	−8.03 (−15.12 to −0.94)	98.00	random
	Liver - tissue	low	5	−12.29 (−17.08 to −7.51)	98.84	random
	Lung - serum	high	7	−1.01 (−1.69 to −0.33)	95.95	random
	Lung - serum	low	6	−1.09 (−1.84 to −0.34)	70.97	random
	Prostate serum	high	5	−2.07 (−3.82 to −0.32)	98.59	random
	Prostate serum	low	2	−0.34 (−3.07 to 2.39)	78.33	random
	Prostate tissue	high	3	−6.25 (−9.71 to −2.78)	98.02	random
	Prostate tissue	low	9	−3.59 (−5.34 to −1.85)	92.34	random
	Stomach serum	high	1	−0.13 (−0.32 to 0.06)		-
	Stomach serum	low	3	−2.09 (−4.14 to −0.04)	97.68	random
	Stomach tissue	high	2	−1.14 (−1.55 to −0.73)	0.00	fixed
	Stomach tissue	low	1	−0.26 (−0.96 to 0.45)		-
	Overall - serum	high	26	−1.30 (−1.72 to −0.88)	96.62	random
	Overall - serum	low	32	−0.92 (−1.3 to −0.54)	96.77	random
	Overall - tissue	high	23	−0.42 (−1.23 to 0.38)	97.38	random
	Overall - tissue	low	36	−2.12 (−2.77 to −1.47)	96.95	random
**Histological type**						
	Gynecological serum	Ovary	1	−0.07 (−0.47 to 0.32)		-
	Gynecological serum	Uterine cervix	1	−0.49 (−0.77 to −0.21)		-
	Head Neck serum	Larynx	4	−1.52 (−2.35 to −0.69)	81.96	random
	Head Neck serum	Oral Cavity	2	−0.95 (−1.38 to −0.53)	0.00	fixed
	Lung - serum	adenocarcinoma	2	0.02 (−1.49 to 1.54)	95.48	random
	Lung - serum	large cell	2	−0.78 (−1.32 to −0.23)	0.00	fixed
	Lung - serum	NSCLC	4	−0.94 (−1.36 to −0.53)	58.87	random
	Lung - serum	small cell	2	−1.42 (−3.05 to 0.21)	88.46	random
	Lung - serum	squamous cell	2	−1.23 (−2.75 to 0.29)	97.01	random
	Lung - tissue	adenocarcinoma	2	−0.89 (−2.96 to 1.19)	92.20	random
	Lung - tissue	large cell	1	−1.22 (−2.22 to −0.23)		-
	Lung - tissue	NSCLC	2	−0.62 (−1.88 to 0.65)	88.90	random
	Lung - tissue	small cell	2	−0.52 (−0.80 to −0.23)	0.00	fixed
	Lung - tissue	squamous cell	2	−0.48 (−2.54 to 1.58)	92.14	random
	Thyroid serum	folicular	2	−1.86 (−4.83 to 1.11)	96.81	random
	Thyroid serum	papillary	2	−1.48 (−4.43 to 1.48)	96.38	random
	Thyroid tissue	folicular	1	0.00 (−0.59 to 0.59)		-
	Thyroid tissue	papillary	1	−0.50 (−1.09 to 0.09)		-

Note low is for Newcastle-Ottava scale <6, high for NOS >6. Random and fixed effect meta-analysis. NSCLC, non-small cell lung cancer, CI, confidence interval.

### Statistical Analyses

To express the differences in serum and tissue concentrations, standardized difference in means (Cohen’s d) was used. To assess heterogeneity across studies, Higgins I^2^ (describes percentage of variability) was calculated [12]. Random effects model meta-analysis was employed when I^2^>50%; otherwise, fixed model was used. Publication bias was evaluated using funnel plots and two-sided Egger tests in groups with >3 studies. Funnel plots of studies with Egger’s test p<0.05 are asymmetric (Table S1). There was performed meta-regression using unrestricted maximum likehood method of studies reporting stage or grade if number of studies with corresponding moderators was >10 [13], [14]. Comprehensive Meta-analysis Version 2 software (Biostat, Englewood, NJ) was used for analysis.

## Results

### Identification of Relevant Studies and their Characteristics

Total of 3201 articles were found. After excluding articles not meeting the criteria (Fig. 1) and duplicates, 114 articles studying 130 tumors were included (several studies studied more tumors, Table S2).

Overall 114 studies including 22737 participants (8584 cases, 14153 controls) were analyzed. From studies reporting age, sex and ethnicity, the mean age was 54.5±12.3 (male) and 49.6±12.2 (female); male were included 49.5% and 51.0% in “tumor” and control groups. Publication date ranged within 1952–2012. Caucasians, Asians, Hispanics, and Afro-Americans were reported in 52%, 45%, 1%, and 2%, respectively. Characteristics of studies are summarized in Table S2. Two clinical, 4 cohort, 6 cross-sectional, and 102 case control studies were included.

### Overall Zinc Level in Sera and Tumors

#### Serum level

**Figure 2:**
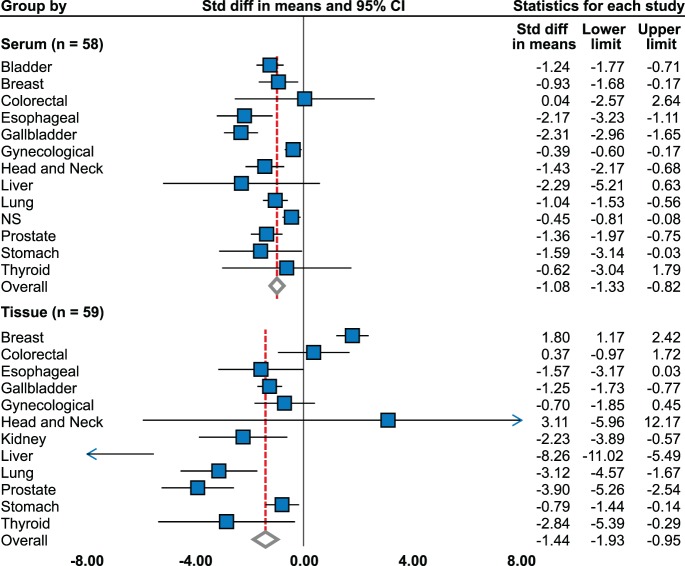
Level of zinc in sera and tissues by tumor type. Summary of individual meta-analyses. For model used and heterogeneity, see Table 1.

As shown on Fig. 2 and Table 1, serum zinc level is significantly decreased in patients with tumors (effect size = −1.08; 95% confidence interval, CI, −1.33 to −0.82) using random effects model of 58 studies (6223 cases, 10364 controls). This is consistent with subgroup of 26 high-quality studies (effect size = −1.30; 95% CI, −1.72 to −0.88). High level of heterogeneity is observed (Higgins I^2^ = 96.71%). Meta-regression did not reveal that stage, grade, and age or publication year affect effect size (Table S1). Six studies analyzed serum zinc level in group of patients with malignant tumor without other specification (516 cases, 3871 controls), and significant decrease was found (Fig. 2) [15]–[20].

#### Tissue level

There was a significant decrease in tissue zinc level using random effects model meta-analysis of 59 studies (2361 cases, 3789 controls) with effect size −1.44 (CI−1.93 to −0.95). However, publication bias was observed at p = 0.01 and no significance found in 24 high-quality studies. Meta-regression did not reveal any moderators to affect global effect size.

### Bladder

Significant decrease of serum zinc level was observed (−1.24; 95% CI, −1.77 to −0.77) using random effects model of two studies [21], [22] (86 cases, 92 controls); both were “high-quality”.

### Breast

#### Serum

**Figure 3:**
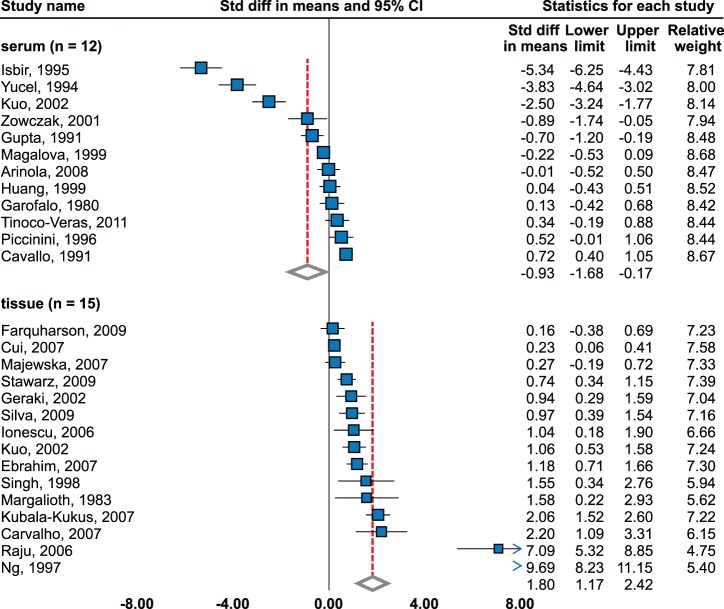
Zinc level in sera and tissues of breast cancer patients. Random effects model meta-analysis. Studies sorted by standardized mean difference.

There was significant decrease (−0.93; 95% CI, −1.68 to −0.17) using random effects model meta-analysis of 12 studies (604 cases, 663 controls) [20], [23]–[33] (Fig. 3, Table 1). However, no significant change was observed in six studies [20], [23], [25], [27], [30], [31] and significantly increased level in one [24]. No publication bias was observed (Begg’s funnel plot was symmetrical, Egger’s 2-tailed test p = 0.086). Subgroup meta-analysis by methodological quality of study revealed significant decrease in nine low-quality studies (− 1.32, CI–2.23 to–0.42) using random effects model, but no significant change in three high-quality studies using fixed effects model [25], [27], [31]. With regard to stage, Yucel *et al.* found no difference between stages [33]; in contrary, studies by Gupta *et al.* and Kuo *et al.* showed a decrease in advanced cancer in comparison to early stages [26] and significantly decreasing trend in relation to stage [29].

#### Tissue level

Significant elevation was determined using random effects model of 15 studies (635 cases, 714 controls), effect size = 1.80 (95% CI, 1.17 to 2.42) [29], [34]–[47]. However, two studies show insignificant changes [42], [46]. The highest levels were observed in study by Ng *et al.*, which include ductal cancers only [35]. Publication bias was observed on p = 0.018. Significant elevation was found in both low- and high- quality studies, while the levels were higher in 9 high-quality studies (2.33; CI, 1.46 to 3.21). Farquharson *et al.* reported significantly decreased tissue zinc concentration in estrogen receptor negative tumors [42]. Kuo *et al.* found no trend [29] in relation to grade, while Farquharson *et al.* showed significant decrease in grade II-III vs. grade I [42].

### Gynecological Tumors (Uterine Corpus, Cervix and Ovarian)

#### Serum

Among ovarian and cervical cancers, there was a significant decrease found (−0.39; CI−0.60 to −0.17) using fixed effects model of three studies (164 cases, 171 controls) [20], [48], [49]. No publication bias was observed. When subgroup meta-analysis by histological type was done, significant decrease was found in cervical [48], insignificant changes in ovarian cancer [49]. There were no significant trends in relation to stage and grade [49].

#### Tissue level

No significant difference between uterine corpus and cervix cancers was determined using random-effects model of 4 studies (80 cases, 123 controls) [37], [48], [50], [51]. No publication bias was determined. Significant decrease was reported in two studies [48], [51]. Results of high-quality studies did not show a significant trend.

### Digestive System Tumors (Esophageal, Stomach, Colorectal, Liver, Gallbladder and Pancreatic Carcinoma)

#### Esophageal, serum

All four studies included in this analysis (93 cases, 80 controls) [52]–[55] show significant reduction (−2.17; 95% CI, −3.23 to 1.11) and it was one of the highest decreases. Publication bias was identified (p = 0.04) and insignificant decrease was observed in two high-quality studies.

#### Esophageal, tissue

No significant change was identified using meta-analysis of three studies (104 cases, 116 controls) [52], [56], [57] due to high variability among studies, even among two high-quality ones [56], [57]. No publication bias was observed.

#### Stomach, serum

Significant decrease was observed (effect size = −1.59; 95% CI, −3.14 to −0.03) without publication bias. However, high variability was present in the four studies (290 cases, 474 controls) [28], [30], [58], [59] and significant decrease was reported in two studies only [28], [59]. No significant change was observed in one high-quality study [58].

#### Stomach, tissue

Significant decrease was determined (− 0.79; 95% CI, −1.44 to −0.14) in three studies (71 cases, 67 controls) [37], [59], [60] and one study showed significant decrease [59]. No publication bias was identified.

#### Colorectal cancer, serum

No significant difference was determined. No study fulfilled criteria of “high-quality”. Five studies were included (313 cases, 216 controls) [28], [30], [61]–[63] and no publication bias was detected. However, two studies showed significant decrease [28], [62], one study showed significant elevation [61], and thus, high serum zinc level variances were associated with colorectal cancer. Of studies reporting stage, one showed significantly decreasing trend on Dukes stage [62], whereas other showed no significant changes related to TNM stage [61].

#### Colorectal cancer, tissue

No significant difference was observed using meta-analysis of seven studies (233 cases, 159 controls) [34], [37], [46], [62], [64]–[66] and no publication bias was observed. None of the high quality studies revealed significant differences. One of the largest variation among tissue levels was observed (effect size = 0.37; 95% CI, −0.97 to 1.72). Two studies showed significant decrease [62], [64] while another two significant elevation [46], [66]. One study including grade did not show significant trend [64].

#### Gallbladder, serum

one high-quality study (30 cases, 30 controls), that showed significant decrease, was identified [67].

#### Gallbladder, tissue

Significant decrease was determined in two studies [68], [69] (39 cases, 40 controls) using fixed effects model (−1.25; 95% CI, −1.73 to −0.77). No study analyzed further classifications.

#### Liver, serum

No significant change was observed in three studies [70]–[72] (149 cases, 121 controls) and no publication bias was observed. Two high-quality studies [71], [72] are in accordance, showing fixed serum zinc level. One study showed no significant trend in relation to stage [70].

#### Liver, tissue

Significant decrease was observed in all 7 studies analysed [70], [73]–[78] (269 cases, 329 controls), effect size = −8.26 (95% CI, −11.02 to −5.49). Two high-quality studies [73], [76] provided consistent results (−8.03; 95% CI, −15.12 to −0.93). Publication bias was observed (p = 0.04).

### Prostate

#### Serum

**Figure 4:**
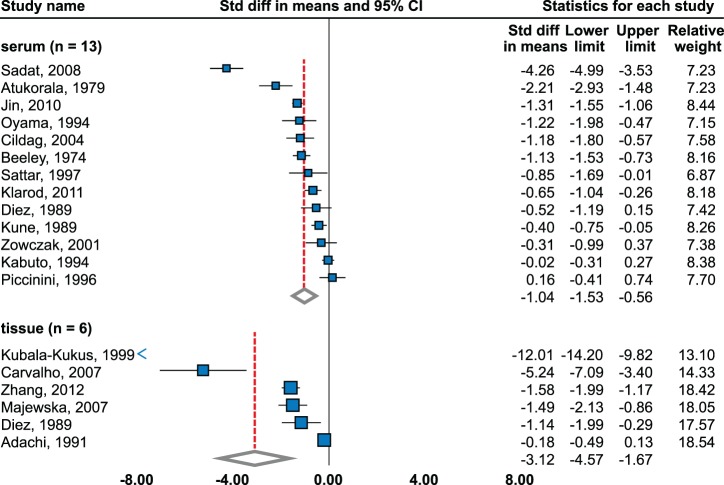
Zinc level in sera and tissues of prostate cancer patients. Random effects model meta-analysis. Studies sorted by standardized mean difference.

Significant decrease was observed (effect size = −1.36; 95% CI, −1.97 to −0.75) using random effects model-meta-analysis of 7 studies [79]–[85] (2985 cases, 3539 controls, Fig. 4).

Insignificant changes were observed in large sample studies – cohort from French SuViMax study (4961 cases and controls) [82] and Multiethnic Cohort study (1175 cases and controls) [85]. Subgroup meta-analysis found significant decrease in high quality studies (−2.07; 95 CI, −3.82 to −0.32). No publication bias was observed.

#### Tissue

Random effects model meta-analysis of 12 studies (240 cases, 226 controls) [86]–[97] detected a significant decrease (effect size = −3.90; 95% CI, −5.26 to −2.54). Only one study showed insignificant decrease [86]. Most significant decrease was observed in study by Guntupalli et al. [89]. High level of publication bias was observed among studies (p = 0.0007). Results are in agreement with more distinct effect size in high-quality studies (−6.03; 95% CI, −9.39 to −2.67). Trend was not observed in one study relating to stage and grade [6].

### Head and Neck

#### Serum

Five studies including cancers of oral cavity [98], [99] and larynx [98], [100]–[102] (159 cases, 228 controls) showed significant decrease (random effects model −1.43; 95% CI, −2.17 to −0.68). No publication bias was observed – all studies, including 2 high-quality ones, showed significant decrease [98], [99]. No significant trend was observed between stage and serum zinc level [100].

#### Tissue

Two studies (45 cases, 27 controls) [103], [104] were included in the analysis. Findings of these studies were contradictory: one showed significant elevation [103] while the other - significant decrease [104]. Random effects model did not show any trend. No significant trend between grade and zinc level was observed in one study [104].

### Thyroid

#### Serum

No significant difference was observed using random effects model of three studies (131 cases, 93 controls) [105]–[107]. One study showed significant elevation [107], the other showed significant decrease [105] and third, ranked as high quality [106], found no significant differences. No publication bias was observed. Subgroup analysis by histological type does not highlight significant difference between papillary and follicular cancer. Medullar carcinoma was not included in meta-analysis.

#### Tissue

Statistically significant decrease was observed using random effects model (effect size = −2.84; 95% CI, −5.39 to −0.29) of three studies (109 cases, 123 controls) [105], [108], [109]. However, one study reported insignificant results [105], another included papillary, follicular, medullar cancers and reticulosarcoma [108] and the third did not specify histological types. No publication bias was present. One study determined no significant difference between papillary and follicular cancers [105].

### Kidney

#### Tissue

Significant decrease was observed using random effects model (−2.23; 95% CI, −3.89 to −0.57) of 4 studies (66 cases, 45 controls). Results agree with 2 high quality studies [37], [110]. All studies showed significant decrease [37], [110]–[112] and no publication bias was detected.

### Lung

#### Serum

**Figure 5:**
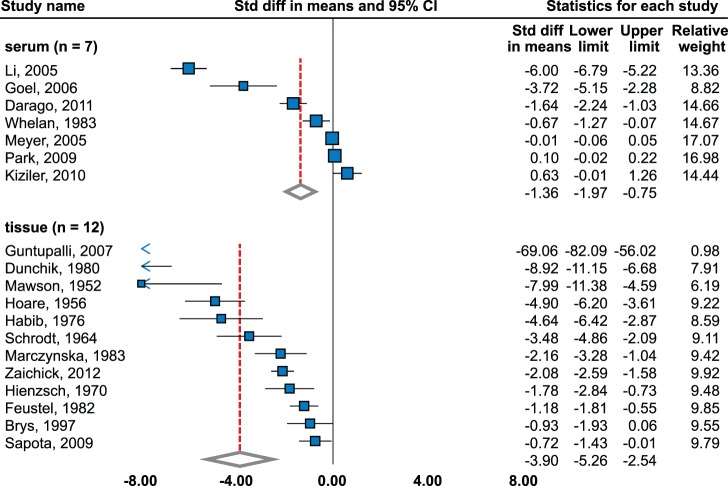
Zinc level in sera and tissues of lung cancer patients. Random effects model meta-analysis. Studies sorted by standardized mean difference.

Significant decrease (−1.04; 95% CI, −1.53 to −0.56) was identified using random effect model of 13 studies [20], [31], [58], [113]–[122] (703 cases, 786 controls, Fig. 5); four of them showed insignificant changes [20], [31], [58], [115]. No publication bias was observed (p = 0.38). Analysis of only high-quality studies provided similar results. Subgroup analysis according to histology detected significant decrease in non-small cell lung cancer using random effects model in four studies. Two studies dealt with histological classification [115], [116]: no significant difference was observed in adenocarcinoma and squamous cell carcinoma using random effects and significant decrease in large cell carcinoma using fixed effects (−0.78; 95% CI, −1.32 to −0.23). serum zinc level and stage was analyzed in two studies [114], [116]. Klarod *et al.* determined significantly lower serum zinc level in advanced compared to low stages [114]. Similarly, descending trend was observed between stages T1, T2, and T3 [116]. Negative correlation between serum zinc level and grade was determined [116].

#### Tissue

Significant decrease was determined using random effects model (−3.12; 95% CI, −4.57 to −1.67) of six studies, all ranked as low-quality [34], [46], [115], [123]–[125] (470 cases, 1820 controls). However, publication bias was observed (p = 0.03). Insignificant change was observed in one study [123]. Significant decrease was determined in small cell lung cancer (−0.52; 95% CI, −0.80 to −0.23) using fixed model and no significant decrease was identified in non-small cell lung cancer using random effects model. No significant trend was observed in squamous and adenocarcinomas. Large cell cancer showed significant decrease in one study [115].

## Discussion

Decreased serum zinc level was found in patients with lung, head and neck, breast, liver, stomach, and prostate cancers. The elevation was not proven in any tumor. More specific zinc patterns are evident in tumors. Unequivocal increase was observed in breast cancer tissue only and decrease in prostatic, liver, lung, and thyroid cancer. The rest of the studied tumors brought ambiguous results, both in serum and tissue zinc levels across the studies. It cannot be confirmed that the serum zinc level does not change except of the abovementioned tumors. Serum and tissue zinc level reduction was evident to certain extent in majority of tumors. Although insignificant differences were found, the analysis indicates that none of the tumors clearly disproves that the zinc levels remained unchanged. Variation of serum zinc level were found in esophageal cancer patients, in cell zinc content in liver cancer and both in serum and tissue zinc level in stomach, colorectal, and thyroid cancers.

Number of studies point to decreasing trend in tumors of higher grades or stages. Nevertheless, meta-regression could not be performed on the majority of tumors due to limited number of studies reporting stage/grade or to inconsistency in the scale used. Regression analysis of all tumors, however, did not show dependence on these parameters. Thus, this meta-analysis fails to explain the sources of high heterogeneity between the studies.

Although serum zinc level decrease in lung, head and neck, and breast carcinomas was shown by meta-analysis, it is unclear, whether hypozincaemia is a consequence of tumor, chronic stress or of a combination of both these effects. Stress, infection or chronic diseases lead to redistribution of zinc(II) between body compartments, and thus reduce zincaemia [126]. In addition, chronic inflammation is a common hallmark of cancer, and thus might be important mechanism of serum zinc level decrease.

The association of tissue zinc level and prostate [6], liver [7], and lung [8] cancers serum zinc level and risk of breast, lung, stomach, and prostate cancers [9] were in scope of several reviews. Decrease in prostate cancer tissue zinc level is well-evidenced [127]. Also review by Zaichick *et al.* show decrease of zinc in prostate cancer tissue as compared with benign hyperplasia [6]. A review by Catalani, focusing on zinc content in lung tumors, is the only to date meta-analysis. However, its results did not allow summarizing the significance of tissue metals. No relationship among tissue zinc level and histotype or stage was found. Zinc decrease in liver cancer tissues were reviewed by Gurusamy *et al.* They declared that meta-analytic approach is impossible because of heterogeneity of analyzed studies. All mentioned reviews concluded that there is poor data agreement between studies determining tissue zinc level. This fact – combined with the low metal concentrations – calls for the standardization of methods. Catalani *et al.* propose standardization of sample collection, storage, and analysis. Previous reviews were performed only on specific tumors, with limited number of studies and/or statistic approaches were missing. Our meta-analytical analysis was done on all identified carcinomas, serum and tissue levels were analyzed together, publication bias was assessed and meta-regression was performed when case sufficient data were present. To reduce selection and publication biases, prospective cohort study with defined conditions separating the influence of inflammation is needed. Interest should be focused on the relation of zinc level in each histological type, stage, and grade.

There are limitations in this study caused by features of individual studies: sample sizes, subjects’ characteristics, sampling, storage and detection methods, and different tumors classification. Serum zinc level has a limited predictive value, because it is particularly intracellular ion and it fluctuates in circadian rhythm.

This meta-analysis shows a decrease of zincaemia in lung, head and neck, and breast carcinoma, increase of tissue zinc in breast cancer and its decrease in prostate, liver, and lung cancers. However, this analysis does not provide conclusive data with regard to stage and grade, and thus does not clarify heterogeneity in values between the studies.

## Supporting Information

Checklist S1 Prisma 2009 checklist.
(DOC)

Table S1 Meta-regression analysis of overall results using mixed effects model (unrestricted maximum likehood). SMD, standardized mean difference.
(XLSX)

Table S2 Source data set extracted from studies used for analysis. Including methodological quality of studies based on Newcastle-Ottawa scale, study design, and information regarding matching cases and controls. NS, not specified, NOS, Newcastle-Ottawa scale, stdev, standard deviation.
(XLSX)
